# A stochastic mathematical model of 4D tumour spheroids with real-time fluorescent cell cycle labelling

**DOI:** 10.1098/rsif.2021.0903

**Published:** 2022-04-06

**Authors:** Jonah J. Klowss, Alexander P. Browning, Ryan J. Murphy, Elliot J. Carr, Michael J. Plank, Gency Gunasingh, Nikolas K. Haass, Matthew J. Simpson

**Affiliations:** ^1^ School of Mathematical Sciences, Queensland University of Technology (QUT), Brisbane, Australia; ^2^ School of Mathematics and Statistics, University of Canterbury, Christchurch, New Zealand; ^3^ Te Pūnaha Matatini, New Zealand Centre of Research Excellence in Complex Systems and Data Analytics, New Zealand; ^4^ The University of Queensland Diamantina Institute, The University of Queensland, Brisbane, Australia

**Keywords:** cancer, melanoma, individual-based model, FUCCI, population dynamics

## Abstract

*In vitro* tumour spheroids have been used to study avascular tumour growth and drug design for over 50 years. Tumour spheroids exhibit heterogeneity within the growing population that is thought to be related to spatial and temporal differences in nutrient availability. The recent development of real-time fluorescent cell cycle imaging allows us to identify the position and cell cycle status of individual cells within the growing spheroid, giving rise to the notion of a four-dimensional (4D) tumour spheroid. We develop the first stochastic individual-based model (IBM) of a 4D tumour spheroid and show that IBM simulation data compares well with experimental data using a primary human melanoma cell line. The IBM provides quantitative information about nutrient availability within the spheroid, which is important because it is difficult to measure these data experimentally.

## 1. Introduction

**Figure 1.**
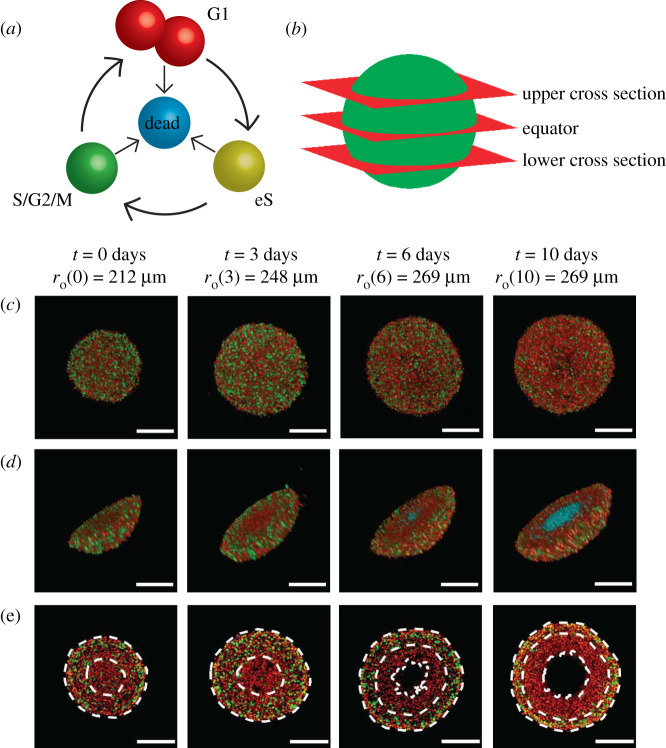
Motivation. (*a*) A schematic of the cell cycle, indicating the transition between different cell cycle phases, and their associated FUCCI fluorescence. Red, yellow and green colouring indicates cells in G1, eS and S/G2/M phase, respectively. (*b*) Locations of the upper cross section, equator and lower cross section. (*c*–*e*) Experimental images of a tumour spheroid using the human melanoma cell line WM793B at days 0, 3, 6 and 10 (after formation) showing. (*c*) Full spheroids, viewed from above, (*d*) spheroid hemispheres and (*e*) spheroid slices, where the cross section is taken at the equator. White dashed lines in (*e*) denote the boundaries of different regions, where the outermost region is the proliferative zone, the next region inward is the G1-arrested region, and the innermost region at days 6 and 10 is the necrotic core. In (*a*) and (*d*), we use cyan colouring for dead cells, which assist in identifying the necrotic core in (*d*). Spheroid outer radii are labelled alongside their corresponding time points, and scale bars represent 200 μm.

*In vitro* tumour spheroids are widely adopted to study avascular tumour growth and drug design [1–3]. Unlike simpler two-dimensional assays, tumour spheroid experiments exhibit heterogeneity within the growing population which is thought to be partly driven by spatial and temporal differences in the availability of diffusible nutrients, such as oxygen [3,4]. Historically, tumour spheroid experiments are analysed using bright-field imaging to measure the spheroid size [5,6], however, this does not reveal information about the internal structure of the spheroid. Since 2008, *fluorescent ubiquitination-based cell cycle indicator* (FUCCI) has enabled real-time identification of the cell cycle for individual cells within growing populations [4,7,8]. Using FUCCI, nuclei of cells in G1 phase fluoresce red, nuclei of cells in S/G2/M phase fluoresce green, and nuclei of cells in early S (eS) phase appear yellow as a result of both red and green fluorescence being active [7] (figure 1*a*). FUCCI provides information about spheroid size and heterogeneity of the cell cycle status (figure 1*c*–*e*). At early times the entire spheroid is composed of freely cycling cells, with a relatively even distribution of FUCCI colours, whereas at intermediate times cells in the central region becomes predominantly red, indicating G1-arrest [4]. Late time growth is characterized by the formation of a central necrotic region, indicated by the absence of fluorescence. FUCCI allows us to identify both the position of individual cells in three spatial dimensions, as well as identifying cell cycle status, leading to the notion of a *four-dimensional (4D) tumour spheroid* [9]. Assuming spherical symmetry, the geometry of 4D spheroids can be characterized by three radii: (i) $r_{o}(t)>0$ is the outer radius; (ii) $r_{a}(t)\geq 0$ is the arrested radius; and, (iii) $r_{n}(t)\geq 0$ is the necrotic radius, with $r_{o}(t)>r_{a}(t)\geq r_{n}(t)$. In figure 1*e*, we see that $r_{n}(t)=0$ for *t* ≤ 3, with the necrotic core forming sometime between *t* = 3 and t=6 days.

Continuum mathematical models of tumour spheroids have been developed, analysed, and deployed for over 50 years [10–15], and these developments have included very recent adaptations of classical models to study tumour spheroids with FUCCI [9,16,17]. Continuum modelling approaches lack the ability to track individual cells within the growing population, and typically neglect heterogeneity and stochasticity. In comparison, individual-based models (IBMs) allow us to study population dynamics in more detail, by keeping track of all individuals and explicitly capturing heterogeneity and stochasticity [18,19]. Over the last 20 years, as computing power has increased at the same time that experimental imaging resolution has improved, there has been an increasing interest in interpreting tumour spheroid experiments using IBMs [20–25], with some studies using these models to focus explicitly on how the balance of cell migration and cell proliferation impacts phenotype selection [26]. Flegg & Nataraj [27] succinctly review mathematical modelling methodologies used to interpret tumour spheroid experiments. While several previous IBMs have the ability to track the cell cycle within individuals [20–25], our aim is to track the cell cycle in a relatively minimal IBM and to quantitatively interpret this information in terms of a new set of 4D tumour spheroid experiments.

In this work, we develop an IBM of 4D tumour spheroid growth with FUCCI. The IBM describes how individual cells migrate, die and progress through the cell cycle to mimic FUCCI. Certain mechanisms in the IBM are coupled to the local availability of a diffusible nutrient. The biological fidelity of the IBM is demonstrated by qualitatively comparing simulation results with detailed experimental images at several cross sections (figure 1*b*). Quantitative data from the model are used to assess the spheroid population distribution, nutrient concentration, and the role of variability in spheroid growth. We extract and quantitatively compare simulation radius estimates with measurements from a series of 4D tumour spheroid experiments using a human primary melanoma cell line (figure 1). Using a careful choice of parameter values, we show that the IBM quantitatively replicates key features of 4D tumour spheroids.

## 2. Methods

Experimental methods are described in electronic supplementary material, §S1.

### 2.1. Individual-based mathematical model

We simulate 4D spheroid growth inside a cubic domain, Ω, of side length *L*, where *L* is chosen to be large enough so that agents do not reach the boundary of the domain during the simulation, but not so large as to incur significant computational overhead (electronic supplementary material, S4.3). Biological cells are represented as discrete agents located at **x**_*n*_(*t*) = (*x*_*n*_(*t*), *y*_*n*_(*t*), *z*_*n*_(*t*)) for *n* = 1, 2, 3, …, *N*(*t*), where *N*(*t*) is the total number of agents at time *t*.

#### 2.1.1. Gillespie algorithm

**Figure 2.**
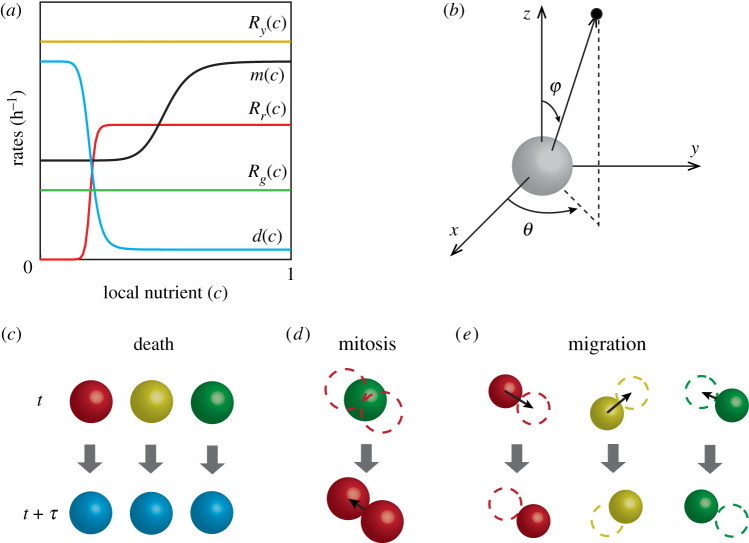
IBM schematic. (*a*) Nutrient-dependent rates (equations (2.1)–(2.5)). (*b*) Random directions for migration and mitosis are obtained by sampling the polar angle *θ*, and the azimuthal angle φ separately. (*c*–*e*) Schematics showing agent-level events; death, mitosis and migration, across a time interval of duration *τ*. (*c*) Any living agent may die, removing it from the simulation. (*d*) An agent located at **x**_*n*_ undergoes mitosis to produce two daughter agents in G1 phase and dispersed a distance of *σ*/2 from **x**_*n*_ in opposite, randomly chosen directions. (*e*) Any living agent can migrate in a random direction with step length μ.

The IBM describes key cellular-level behaviours; namely cell cycle progression and mitosis, cell motility, and cell death, as discrete events simulated using the Gillespie algorithm [28]. Each agent has an allocated rate of cell cycle progression, dependent on its cell cycle status and the local nutrient concentration (figure 2*a*). Agents in each phase of the cell cycle are coloured according to FUCCI, with G1 agents coloured red, eS agents coloured yellow, and S/G2/M agents coloured green.

We make the natural assumption that biological cells require access to sufficient nutrients to commit to entering the cell cycle. Therefore, the red-to-yellow transition rate, *R*_*r*_(*c*), depends on the local nutrient concentration, *c*(**x**, *t*) (figure 2*a*). Once an agent has committed to entering the cell cycle, we assume the yellow-to-green transition takes place at a constant rate *R*_*y*_, and the green-to-red transition, which involves mitosis, occurs at a constant rate *R*_*g*_ (figure 2*a*).

The rates of agent migration and death, *m*(*c*) and *d*(*c*), respectively, are assumed to depend on the local nutrient concentration. When an agent dies, it is removed from the simulation and we record the location at which the death event occurs (figure 2*c*). When an agent moves or undergoes mitosis (figure 2*d*,*e*), a random direction in which the agent will migrate, or its daughter agents will disperse, is chosen (figure 2*b*). For an agent undergoing mitosis, the first daughter agent is placed a distance *σ*/2 along the randomly chosen direction, and the second daughter agent is placed at a distance *σ*/2 in the opposite direction, leaving the two daughter agents dispersed a distance of *σ* apart, where we set *σ* to be equal to a typical cell diameter [29] (figure 2*d* and table 1). When migrating, agents are displaced a distance μ along the randomly chosen direction (figure 2*e*). Similar to the dispersal, we simulate migration by taking the step length μ to be a typical cell diameter.

**Table 1.** IBM parameter values.

parameter name	symbol	value	source
*numerical parameters*			
initial number of agents	*N*(0)	30 000	experimental measurement (electronic supplementary material, S7)
initial number of red agents	*N*_r_(0)	20 911	assumption (electronic supplementary material, S8)
initial number of yellow agents	*N*_y_(0)	995	assumption (electronic supplementary material, S8)
initial number of green agents	*N*_g_(0)	8094	assumption (electronic supplementary material, S8)
domain length	*L*	4000 μm	numerical experiments (electronic supplementary material, S4.3)
initial spheroid radius	$r_{o}(0)$	245 μm	experimental measurement
dispersal distance	*σ*	12 μm	assumption (electronic supplementary material, S5)
migration distance	μ	12 μm	assumption (electronic supplementary material, S5)
simulation time	*T*	240 h	experimental measurement
*per capita agent rates*			
maximum G1–eS transition rate	*R*_*r*_	0.047 h^–1^	experimental measurement (electronic supplementary material, S6)
constant eS–S/G2/M transition rate	*R*_*y*_	0.50 h^–1^	experimental measurement (electronic supplementary material, S6)
constant S/G2/M–G1 transition rate (mitosis)	*R*_*g*_	0.062 h^–1^	experimental measurement (electronic supplementary material, S6)
maximum death rate	*d*_max_	2 h^–1^	assumption
minimum death rate	*d*_min_	0.0005 h^–1^	assumption
maximum migration rate	*m*_max_	0.12 h^–1^	assumption
minimum migration rate	*m*_min_	0.06 h^–1^	assumption
hill function index for arrest	*η*_1_	5	assumption
hill function index for migration	*η*_2_	5	assumption
hill function index for death	*η*_3_	15	assumption
*nutrient parameters*			
number of nodes	*I*^3^	201^3^	assumption (electronic supplementary material, S4.4)
steady-state solution interval	t*	1 h	assumption (electronic supplementary material, S4.4)
consumption-diffusion ratio	*α*	0.15 μm cell^–1^	assumption
critical arrest concentration	*c*_a_	0.4	assumption
critical migration concentration	*c*_m_	0.5	assumption
critical death concentration	*c*_d_	0.1	assumption

We specify the agent cycle progression rates,

2.1 $$R_{r}(c)=R_{r}\frac{c^{\eta_{1}}}{c_{a}^{\eta_{1}}+c^{\eta_{1}}},$$

2.2 $$R_{y}(c)=R_{y},$$

2.3 $$R_{g}(c)=R_{g}$$

2.4 $$m(c)=(m_{\max}-m_{\min})\frac{c^{\eta_{2}}}{c_{m}^{\eta_{2}}+c^{\eta_{2}}}+m_{\min}$$

2.5 $$d(c)=(d_{\max}-d_{\min})\left(1-\frac{c^{\eta_{3}}}{c_{d}^{\eta_{3}}+c^{\eta_{3}}}\right)+d_{\min},$$

where *c*(**x**_*n*_, *t*) ∈ [0, 1] is the non-dimensional nutrient concentration at the location of the *n*th agent; *R*_*r*_ > 0 is the maximum red-to-yellow transition rate; *m*_max_ > *m*_min_ ≥ 0 are the maximum and minimum migration rates, respectively; *d*_max_ > *d*_min_ ≥ 0 are the maximum and minimum death rates, respectively; *η*_1_ > 0, *η*_2_ > 0 and *η*_3_ > 0 are Hill function indices; and *c*_a_ > 0, *c*_m_ > 0 and *c*_d_ > 0 are the inflection points of *R*_*r*_(*c*), *m*(*c*) and *d*(*c*), respectively (figure 2a).

#### 2.1.2. Nutrient dynamics

We make the simplifying assumption that cell migration, death and progression through the cell cycle are regulated by a single diffusible nutrient, such as oxygen [4,10,12]. The spatial and temporal distribution of nutrient concentration, *C*(**x**, *t*), is assumed to be governed by a reaction–diffusion equation

2.6 $$\frac{\partial C}{\partial t}=D\nabla^{2}C-\kappa Cv,\;\text{in }\Omega ,$$

with diffusivity *D* > 0 [μm^2^ h^−1^], and consumption rate *κ* > 0 [μm^3^ (h cells)^–1^], and where *v*(**x**, *t*) ≥ 0 [cells μm^–^^3^] is the density of agents at position **x** and time *t*. The source term in equation (2.6) describes the consumption of nutrient at a rate of *κ* [μm^3^ (h cells)^–1^]. To solve this reaction–diffusion equation, we set *v*(**x**_*i*,*j*,*k*_,*t*) = *N*_*i*,*j*,*k*_/*h*^3^, where *N*_*i*,*j*,*k*_ is the number of agents within the control volume surrounding the node located at (*x*_*i*_, *y*_*j*_, *z*_*k*_) and *h*^3^ is the volume of the control volume. On the boundary, ∂Ω, we impose *C* = *C*_b_, where *C*_b_ is some maximum far-field concentration.

Our experiments lead to spheroids of diameter 500–600 μm over a period of 10 days after spheroid formation (figure 1) (14 days after seeding). Since these length and timescales are clear, we leave the independent variables **x** and *t* in equation (2.6) as dimensional quantities. By contrast, spatial and temporal variations of *C*(**x**, *t*) are very difficult to measure during spheroid growth, so we non-dimensionalize the independent variable *c*(**x**, *t*) = *C*(**x**, *t*)/*C*_b_, giving

2.7 $$\frac{\partial c}{\partial t}=D\nabla^{2}c-\kappa cv,\;\text{in }\Omega .$$

with *c* = 1 on ∂Ω, and *c*(**x**, *t*) ∈ [0, 1].

Typically, the timescale of nutrient diffusion is much faster than the timescale of spheroid growth [10]. Consequently, we approximate equation (2.7) by

2.8 $$0=\nabla^{2}c-\alpha cv,\;\text{in }\Omega ,$$

where *α* = *κ*/*D* > 0 [μm cell^–1^]. Therefore, we describe the spatial and temporal distribution of nutrients by solving equation (2.8) repeatedly during the simulation. This quasi-steady approximation is computationally convenient, as we describe later. We solve equation (2.8) with a finite volume method on a uniform structured mesh (electronic supplementary material, S4) with node spacing *h*.

The IBM treats cells as point particles, which we call agents. Interactions between agents are modelled implicitly by specifying rates of migration and cell cycle progression that depend upon the local nutrient concentration. As we will show later, this very simple framework replicates our experimental observations reasonably well, however, we acknowledge there are other ways to treat agent–agent interactions, including lattice-based hard-core exclusion [29] and lattice-free methods for minimizing [30] or avoiding agent overlap [23,24]. Other options for modelling agent–agent interactions include direct simulation of agent adhesion or repulsion [31]. Here, our modelling philosophy is to work with the simplest possible biologically plausible model that can describe the key features of interest. Therefore, we deliberately avoid these additional mechanisms in our IBM framework.

### 2.2. Simulation algorithm

We simulate spheroid growth by supposing the spheroid initially contains *N*(0) agents distributed uniformly within a sphere of radius $r_{o}(0)>0\;[\mu m]$. While it is experimentally relevant to assume the population is spherically symmetric at *t* = 0, this assumption is not necessary, and we will discuss this point later. The proportion of agents chosen to be red, yellow, or green at *t* = 0 can be selected arbitrarily, but we choose these proportions so that the internal structure and composition of the *in silico* spheroids are consistent with our *in vitro* measurements. We achieve this by choosing the initial numbers of red, yellow, and green agents, *N*_r_(0), *N*_y_(0) and *N*_g_(0), respectively, noting that *N*(0) = *N*_r_(0) + *N*_y_(0) + *N*_g_(0) (electronic supplementary material, S8). The most appropriate timescale for individual cell-level behaviour is hours, however, spheroid development takes place over 10 days, so we will use a mixture of timescales to describe different features of the experiments and simulations as appropriate. We simulate spheroid growth from *t* = 0 to *t* = *T* h, updating the nutrient concentration at *M* equally spaced points in time. This means that the nutrient concentration is updated at intervals of duration t* = *T*/*M* [h]. The accuracy of our algorithm increases by choosing larger *M* (smaller t*), but larger *M* decreases the computational efficiency. We explore this trade-off and find that setting t* = 1 h is appropriate (electronic supplementary material, S4.4). When equation (2.8) is solved for *c*(**x**, *t*), the value of *c*(**x**_*n*_, *t*) at each agent is calculated using linear interpolation. These local nutrient concentrations are held constant for each agent while resolving all the various agent-level events (cycling and proliferation, migration and death) from time *t* to time *t* + t*. After resolving the appropriate agent-level events, we update the agent density before updating the nutrient profile again. Pseudo-algorithms for the IBM are provided (electronic supplementary material, S9), and code to reproduce key results is available on GitHub https://github.com/ProfMJSimpson/4DFUCCI.

### 2.3. IBM image processing

To estimate $r_{o}(t)$, $r_{a}(t)$ and $r_{n}(t)$, we apply methods described in [16,32,33] to the IBM output. Briefly, we import the agent locations from a particular cross section, and map these locations to an (*L* + 1) × (*L* + 1) pixel image, increase the size of the agents to 12 pixels in diameter, and use edge detection to identify and estimate $r_{o}(t)$, $r_{a}(t)$ and $r_{n}(t)$ (electronic supplementary material, S2). This procedure adapts the image processing approach for the experimental images so that it is applicable to the synthetic results from the IBM.

## 3. Results and discussion

We now compare and analyse images and measurements from a range of *in vitro* experiments and *in silico* simulations. All experiments use the WM793B melanoma cell line, which takes approximately 4 days to form spheroids after the initial seeding in the experiments [17]. This means that t=0 days corresponds to 4 days after seeding to give the experimental spheroids sufficient time to form. Snapshots from the IBM correspond to a single realization, however, time-series data from the IBM are reported by simulating 10 realizations of the IBM and then averaging appropriate measurements across the 10 simulations. While we take great care to analyse the simulation images and the experimental images using the same image-processing algorithm, the format of the experimental images is different to the format of the images produced by the IBM, and we do not explicitly account for these differences.

### 3.1. Parameter values

Table 1 summarizes the parameter values. While some parameters are based on separate, independent two-dimensional experimental measurements (electronic supplementary material, S5, S6) or measurements directly from the spheroids where possible (electronic supplementary material, S7), other parameters are chosen based on a series of numerical screening tests (electronic supplementary material, S4). We will return to discuss other options for parameter choices later.

### 3.2. Qualitative comparison of experiments and simulations

**Figure 3.**
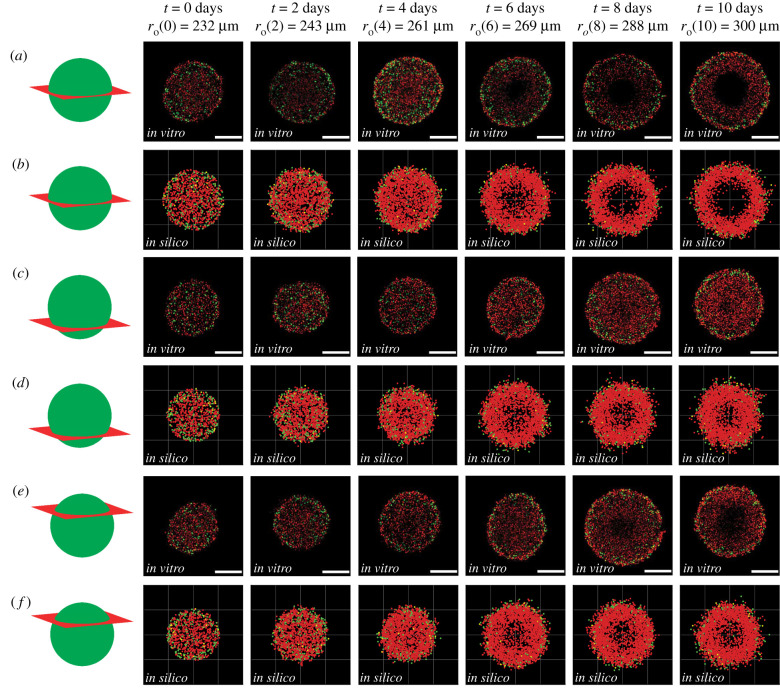
Comparison of *in vitro* and *in silico* 4D spheroids. Experimental results (*a*,*c*,*e*) are compared with simulation results (*b*,*d*,*f*) by examining 2D slices at the equator, lower and upper cross section, respectively. Agent colour (red, yellow, green) corresponds to FUCCI labelling (G1, eS, S/G2/M). Schematics in the left-most column indicate the location of the 2D cross section. The images are taken at (*a*–*b*) the equator, (*c*–*d*) the lower cross section, and (*e*–*f*) the upper cross section. Experimental spheroid radii at the equator are labelled at each time point, and scale bars represent 200 μm.

We now qualitatively compare images of *in vitro* (figure 3*a*,*c*,*e*) and *in silico* (figure 3*b*,*d*,*f*) spheroids by imaging various cross sections at different locations, including the equator (figure 3*a*,*b*), the lower cross section (figure 3*c*,*d*), and the upper cross section (figure 3*e*,*f*). We use definitions in electronic supplementary material, §S1 (Confocal imaging) to identify the lower and upper cross sections in both the experimental and simulation images. While previous studies have often compared model predictions with experimental observations at a single cross section [17,22], we aim to provide more comprehensive information about the internal structure of the spheroid by making comparisons at multiple locations.

At the beginning of the experiment, in all cross sections (*in vitro* and *in silico*), we see the population is relatively uniform, with an even distribution of colours, suggesting the entire spheroid is composed of freely cycling cells. At *t* = 2 and t=4 days, however, we begin to see the development of heterogeneity within the growing *in vitro* and *in silico* populations, with those cells and agents at the centre of the growing spheroid predominantly red, indicating G1-arrest. By t=4 days, we see the value of comparing different cross sections, since the G1-arrest is clear in the centre of the equatorial cross section, but there is no obvious heterogeneity present across either the upper or lower cross sections. Similarly, by t=6 days, we see the formation of a necrotic core in the equatorial cross section, but this is not present at either the upper or lower cross sections. By *t* = 8 and *t* = 10 days, the spheroid has developed into a relatively complicated heterogeneous structure; the outer spherical shell contains freely cycling cells, the intermediate spherical shell contains living G1-arrested cells, and the internal region does not contain any fluorescent cells.

Overall, the qualitative match between the IBM and the experiment confirms that the IBM captures both the macroscopic growth of the entire spheroid, as well as the emergent spatial and temporal heterogeneity. We now build on this preliminary qualitative information by comparing quantitative measurements of growth of the spheroid.

### 3.3. Spheroid structure and nutrient profiles

**Figure 4.**
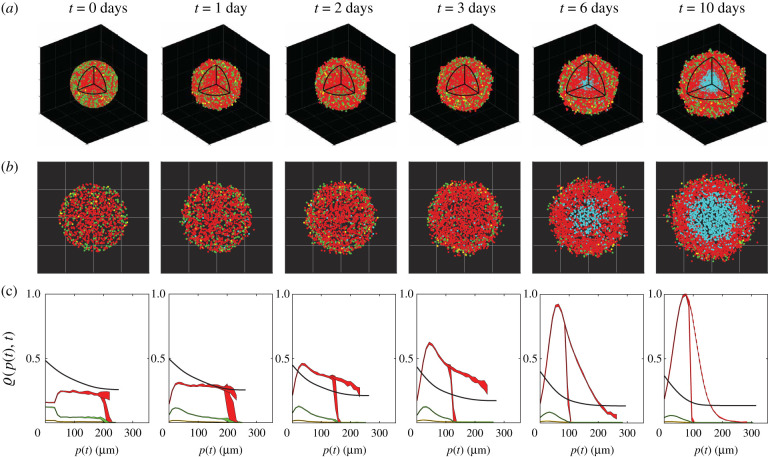
Typical IBM simulation, showing: (*a*) visualizations of *in silico* spheroids including dead agents (cyan) and (*b*) cross sections through the spheroid equator with dead agents. (c) Relative concentrations ϱ(*p*, *t*) of nutrient (black) and cycling red, yellow and green agents (coloured appropriately), based on distance from the periphery $p(t)=r_{o}(t)-r$, averaged over 10 identically prepared simulations. The dashed red line shows the relative density of arrested red agents, also averaged over 10 simulations with identical initial conditions. For nutrient, ϱ(*p*, *t*) = *c*. For agents, ϱ(*p*, *t*) is the relative agent density (electronic supplementary material, S10). Shaded areas represent plus or minus one standard deviation about the mean, and are non-zero as a consequence of stochasticity in the model, even though the 10 simulations start with identical populations and radii.

Given the ability of the IBM to capture key spatial and temporal patterns of spheroid growth, cell cycle arrest, and cell death throughout the spheroid, we now demonstrate how to take these preliminary simulations and extract detailed quantitative data that would be difficult to obtain experimentally. Figure 4*a* shows a typical IBM simulation during the interval where we observe the development of internal structure. For clarity, we plot the locations of all living agents as in figure 3 together with the locations at which agents die, which is difficult to estimate experimentally, but is straightforward with the IBM. Each spheroid in figure 4*a* is shown with an octant removed to highlight the development of the internal structure, and for further clarity we show equatorial cross sections in figure 4*b*.

To quantify the internal spheroid structure we simulate 10 identically prepared realizations of the IBM and extract averaged quantitative data that are summarized in figure 4*c* (electronic supplementary material, S10). These data include plotting the non-dimensional nutrient concentration, *c*(**x**, *t*), and various normalized agent densities, ϱ(*p*(*t*), *t*), as a function of distance from the spheroid periphery, $p(t)=r_{o}(t)-r$, where *r* is the distance from the spheroid centre. Hence, *p*(*t*) = 0 corresponds to the spheroid periphery, and $p(t)=r_{o}(t)$ corresponds to the spheroid centre. This representation of internal spheroid structure is made by assuming that the growing population remains spherically symmetric, which is a reasonable assumption since our initial condition and spheroid growth is spherically symmetric (figure 4*a*). Each density profile is normalized relative to the maximum value of all agent densities across all time points, so that we can compare how the density of the various subpopulations of agents and nutrient are distributed (electronic supplementary material, S10). Using the IBM we are able to describe the spatial and temporal densities of living agents in various phases of the cell cycle (G1, eS and S/G2/M) as well as G1-arrested agents. We plot each density profile as a function of the distance from the periphery as this allows us to compare various profiles as the size of the spheroid increases [9,34].

Averaged relative agent density profiles from the IBM provide quantitative information that cannot be easily obtained from experimental observations. Initially, we see the relatively evenly distributed G1, eS and S/G2/M populations become rapidly dominated by agents in G1 phase, which then form an obvious inner-most arrested region by about *t* = 2 days. During the interval 3 < *t* < 6 days, we observe rapid growth in the arrested population. During the later interval, 6 < *t* < 10 days, we see the formation of a clear necrotic core. These results indicate the spatial and temporal role of stochasticity, with the variability most evident in the G1 and arrested G1 populations at early and intermediate times. Plotting the relative agent densities in this way provides a simple approach to interpret the spatial and temporal organization of cell cycle status within the growing spheroid and visualizing the agent densities together with the non-dimensional nutrient concentration is particularly useful when this kind of information cannot be easily obtained experimentally. In particular, it is technically challenging to measure absolute concentrations of nutrient profiles during these experiments [15,35,36] and so we now focus on visualizing the nutrient concentration profile that drives this heterogeneity.

**Figure 5.**
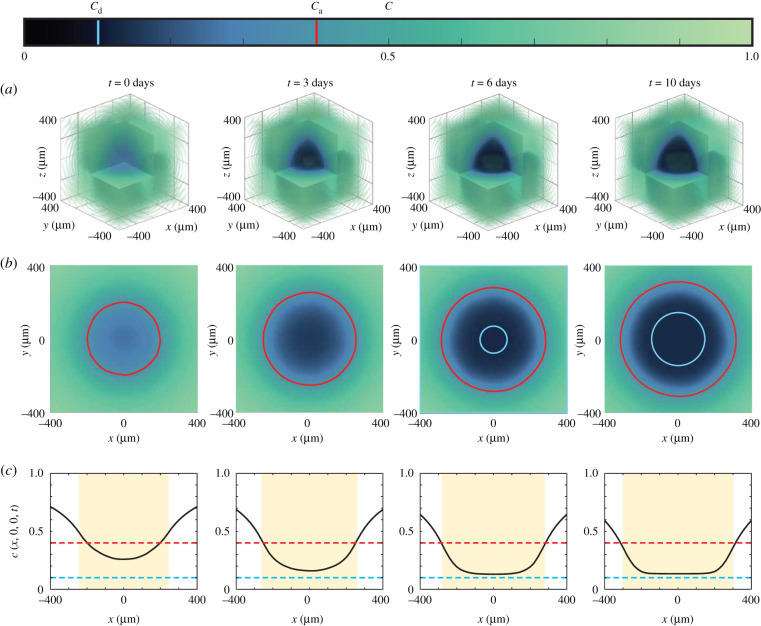
Nutrient concentration profiles (*a*) in three spatial dimensions, (*b*) at the equator *z* = 0, with the arrest critical level *c*_a_ shown in red, and the size of the necrotic region in white. (*c*) Nutrient profiles along the midline *y* = *z* = 0, where the shaded region represents the size of the spheroid, and the red and cyan lines are the critical levels for arrest and death, *c*_a_ and *c*_d_ respectively. The colour bar corresponds to the profiles in (*a*–*b*), and denotes the values *c*_a_ (red) and *c*_d_ (cyan).

Results in figure 5 show spatial and temporal patterns in the nutrient profile, *c*(**x**, *t*), for a typical IBM simulation from figure 4. Figure 5*a* shows the three-dimensional evolution of *c*(**x**, *t*), with the colour bar highlighting the death and arrest thresholds, *c*_d_ and *c*_a_, respectively. These three-dimensional plots show the depletion of nutrient over time in the central region of the spheroid, leading to strong spatial gradients of nutrient concentration near the edge of the growing spheroid. Profiles in figure 5*b* show the nutrient profile at the equatorial plane with the *c*(*x*, *y*, 0) = *c*_a_ contour (red) and the approximate size of the necrotic core (cyan) superimposed. Simplified one-dimensional profiles of *c*(**x**, *t*), along **x** = (*x*, 0, 0), are shown in figure 5*c*, where the diameter of the growing spheroid ($-r_{o}(t)<x<r_{o}(t)$) is shaded in yellow. Again, these simplified cross sections illustrate how nutrient consumption leads to the formation of spatial nutrient gradients near the outer radius of the growing spheroid. Overall, a key strength of the IBM is the ability to extract agent-level information (figure 4) as well as information about the nutrient distribution (figure 5), whereas experimental studies typically report cell-level data without explicitly showing nutrient-level information [4,6].

**Figure 6.**
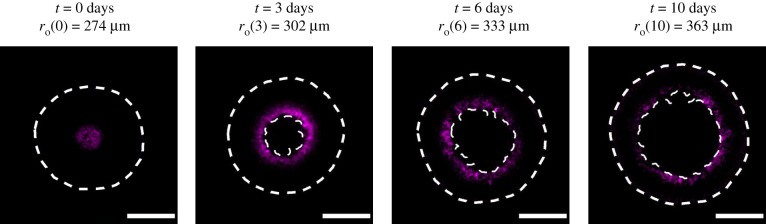
Spheroids stained for hypoxia at 0, 3, 6 and 10 days after spheroid formation, imaged at the spheroid equator. Hypoxia-positive staining fluoresces magenta, and white dashed lines denote $r_{o}(t)$ and $r_{n}(t)$, detected with image processing, to contextualize the regions of hypoxia. For clear visualization, we label the outer radii of the spheroid with the corresponding days. Image intensity was adjusted for visual purposes, and scale bar corresponds to 200 μm.

While it is very difficult to measure the spatial and temporal distribution of diffusible nutrient experimentally in the growing spheroid, it is possible to indirectly examine our assumption that spatial and temporal differences in cell cycle status are partly driven by the availability of oxygen. Figure 6 shows a series of spheroids stained with pimonidazole and pimonidazole-detecting antibodies, which indicate hypoxia [37]. In this series of images, we see evidence of hypoxia staining in the central region of the spheroid at *t* = 0, with persistent hypoxia staining adjacent to the necrotic core at later times. These results support our hypothesis that spatial and temporal differences in nutrient availability correspond with spatial and temporal differences in cell cycle status, and in this case the pimonidazole staining suggests that oxygen availability plays a role in the development of heterogeneity within the growing population. While this observation is consistent with our IBM, it does not rule out the possibility of multiple diffusible signals acting in unison, and we will discuss this possibility later.

### 3.4. Role of variability

Experimental images (figures 1, 3 and 6) suggest that spheroid development is variable, as we see spheroids of different diameters at the same time points. One of the limitations of relying on experimentation alone is that it can be difficult to quantify the importance of different sources of variability, whereas this can be assessed very simply with the IBM. For example, we can simulate multiple spheroids that start from precisely the same initial condition to quantify the variability that arises due to the stochastic growth process, or we can deliberately introduce variability into the initial composition of the spheroid to explore how this variability evolves during spheroid growth for a suite of simulated spheroids.

**Figure 7.**
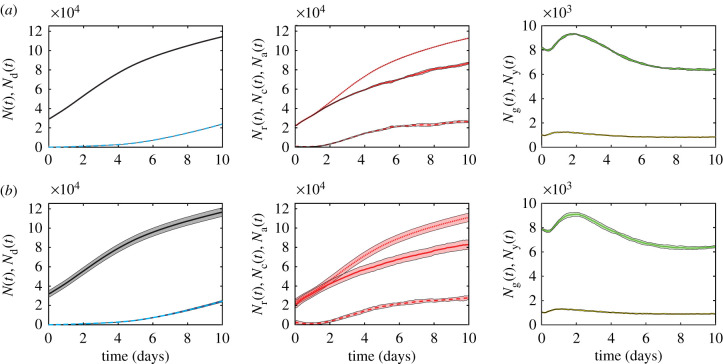
Modelling results for the population growth of different spheroid populations, averaged over 10 simulations with (*a*) identical initial conditions for each realization and (*b*) introduced experimental variability in initial spheroid radius and population, with the agent density held constant and initial radius $r_{o}(t)\in [232.75,235.47,238.97,242.19,244.89,247.76,247.93,251.23,251.48,260.13]\;\mu$m. In each row, left: living (black) and dead (cyan dashed) populations, *N*(*t*) and *N*_d_(*t*), respectively, centre: arrested red (dashed), cycling red (solid) and total red (dotted) populations, *N*_a_(*t*), *N*_c_(*t*) and *N*_r_(*t*), respectively, and right: yellow and green populations, *N*_y_(*t*) and *N*_g_(*t*), respectively. Shaded areas represent plus or minus one standard deviation. Initial subpopulations in each simulation in both (*a*) and (*b*) are variable, as initial cell cycle status is assigned randomly (electronic supplementary material, S8), and so the initial subpopulations in (*b*) also naturally vary with the overall initial population, *N*(0).

Simulation data in figure 7*a* show the temporal evolution of various agent subpopulations, including the total number of living agents, dead agents, G1, eS, S/G2/M and G1-arrested agents. Each profile shows the mean number of agents obtained by simulating 10 identically initialized spheroids with $r_{o}(0)=245\;\mu m$, which matches the average spheroid diameter at *t* = 0 days in the suite of *in vitro* experiments. The variability in these profiles is quantified by calculating the sample mean and sample standard deviation and shading the region corresponding to the sample mean plus or minus one sample standard deviation, and we see that, at this scale, the variability is barely noticeable. By contrast, results in figure 7*b* show equivalent data from a suite of simulations where the initial density of agents in the spheroid is held constant, but the initial radius of the 10 simulated spheroids is deliberately varied to mimic the observed variability in our experiments. The initial radius in each simulation corresponds to one of 10 particular experimental measurements (figure 7). The mean of these 10 initial radii measurements is ${\bar{r}}_{o}(0)=245\;\mu m$, which is precisely the same as the initial radii for the simulations in figure 7*a*. Comparing results in figure 7*a*,*b* shows that the average population profiles are very similar, but the variability is strikingly different. This exercise shows that quantifying the variability in spheroid size at the beginning of the experiment is the key to understanding and predicting the variability in spheroid composition and size at the end of the experiment. The clear differences we see in the variability between results in figure 7*a*,*b* confirms that 10 simulations is sufficient to explore the role of variability at the beginning of the experiment. It is also interesting to note that these simulation results are consistent with our previous observations. For example, the *in vitro* spheroids in figure 3 have $r_{o}(0)=232\;\mu$m and we see that it takes until *t* = 6 days for a clear necrotic core to form in the equatorial cross section. By contrast, the spheroid in figure 6 is larger with $r_{o}(0)=274\;\mu m$ and we see a clear necrotic core at t=3 days. This highlights the importance of taking great care with measurements at the beginning of the experiment [17].

### 3.5. Quantitatively matching experimental and mathematical spheroids

**Figure 8.**
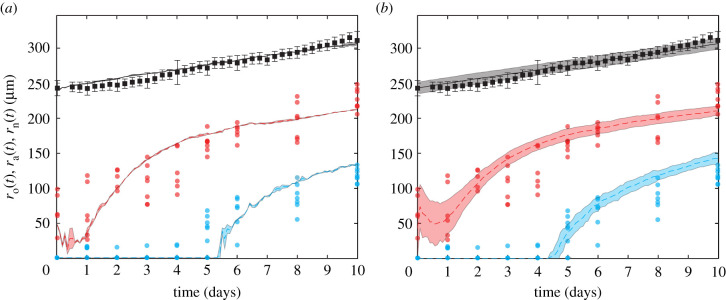
Comparison of computational estimates of $r_{o}(t)$ (black), $r_{a}(t)$ (red) and $r_{n}(t)$ (cyan) with experimental data. The experimental data (dots) are compared with (*a*) simulations with each run starting with an identical parameter set and (*b*) simulations with variations of the initial spheroid radius and population, with each initial radius selected from experimentally measured radii at *t* = 0 days and agent density kept constant. Computational results are the average of 10 simulations, and error regions represent plus or minus 1 s.d. The initial subpopulations vary in both (*a*) and (*b*), due to randomly assigning cell cycle status (electronic supplementary material, S8). In (*b*), we also naturally see higher variations in each subpopulation initially, due to explicitly including initial population variability, which in turn induces variability in $r_{a}(0)$.

Results in figure 8 compare the temporal evolution of $r_{o}(t)$, $r_{a}(t)$ and $r_{n}(t)$, from our suite of experiments and simulations. The data in figure 8 show the value in working with a stochastic model since the experimental measurements are quite variable, with estimates of $r_{a}(t)$ and $r_{n}(t)$ more variable than estimates of $r_{o}(t)$. This difference in variability is because we measure $r_{o}(t)$ automatically every 6 h, whereas measurements of $r_{a}(t)$ and $r_{n}(t)$ require manual harvesting, fixing and imaging, and so we obtain daily measurements only.

Similarly to §3.4, we compare experimental results of average data in simulations with and without explicit variability at *t* = 0. The experiment–IBM comparison in figure 8*a* corresponds to the case where we simulate 10 identically prepared realizations of the IBM, where each simulated spheroid has the same initial radius $r_{o}(0)=245\;\mu m$, and we see that the average simulation results capture the average trends in the experimental measurements well, but the IBM simulations do not capture observed variability in the evolution of $r_{a}(t)$ or $r_{n}(t)$. By contrast, the experiment–IBM comparison in figure 8*b*, where we deliberately mimic the experimental variability at *t* = 0, captures both the average experimental trends and variability in the experimental data quite well. Again, the difference between results in figure 8*a*,*b* suggests that accurately incorporating the initial variability in the experimental data is critical if we wish to capture the observed variability during the experiments with the IBM.

Data in figure 8 show that $r_{o}(t)$ increases approximately linearly, whereas the development of the internal structure is more complicated, with $r_{a}(t)$ initially decreasing for the first day before growing at a similar rate as $r_{o}(t)$. The necrotic core does not form until approximately *t* = 4 days. While our IBM-experimental comparison in figure 8 suggests that the IBM can quantitatively capture experimental trends, we have obtained this match with a careful choice of parameters without undertaking a more rigorous parameter estimation exercise [38]. The sensitivity of the IBM predictions to the parameter values is briefly explored in the electronic supplementary material, S11.

## 4. Conclusion and future work

We developed an IBM that simulates 4D tumour spheroids with FUCCI. IBM simulations show we can successfully reproduce qualitative and quantitative patterns of spatial and temporal differences in cell cycle status observed experimentally. This heterogeneity is driven by spatial and temporal variations in nutrient availability, which we model using a reaction–diffusion equation.

An important advantage of the IBM is our ability to extract and describe measurements that are difficult to obtain *in vitro*. In particular, we show how to visualize both the growing population within the spheroid together with the associated spatial patterns of nutrient concentration over time. Furthermore, the IBM makes it very simple to explore how various features contribute to the overall variability in spheroid development, and we find that relatively small variations in the initial size of the spheroid lead to relatively pronounced differences in spheroid size and composition at later times [17]. We conclude our investigation by showing that we can quantitatively match the spatial and temporal development of a series of *in vitro* 4D spheroids using the WM793B human primary melanoma cell line with a careful choice of parameters. We anticipate that tumour spheroids formed with different cell lines will be able to be simulated with our IBM, but will require different parameter values.

Overall, our modelling philosophy is always to work with the simplest possible mechanisms required to capture our experimental observations. Naturally, this means that there are many ways that the IBM can be extended. For example, here we make the simple assumption that spheroid growth is regulated by a single diffusible nutrient, which seems appropriate for our data. If, however, experiments show that it is important to consider multiple nutrients in unison, our IBM framework can be extended to deal with this. Similarly, we focus on symmetric spheroid growth to be consistent with our experiments, but it is straightforward to relax this assumption by specifying a different initial arrangement of agents, or by allowing asymmetric nutrient delivery by, for example, a blood vessel as we explore in electronic supplementary material, S12. Another point that could be revisited is that we implement the simplest possible cell migration mechanism where the direction of motion is random. While this assumption appears reasonable for our data, it is possible to bias the migration in response to either the nutrient concentration, the gradient of the nutrient concentration, or the density of agents by explicitly describing agent-to-agent interactions [30]. Each of these potential extensions could be incorporated into our IBM framework with the aim of potentially increasing the biological fidelity of the model. However, we caution against this simplistic approach since these mechanisms also increase the number of unknown model parameters required for simulation. To minimize issues with parameter identifiability, we prefer to work with a minimal model [38]. If, however, future experimental measurements indicate that our minimal assumptions need revising, our IBM framework is sufficiently flexible to incorporate such extensions. Another option for future refinement is to conduct a more thorough parameter estimation exercise [38]. Here, we carefully chose parameters so that data from the IBM matches our experimental data, but future analysis could include a more rigorous assessment of parameter estimation, and we leave this for future consideration.

## Data Availability

All data are available on GitHub at https://github.com/ProfMJSimpson/4DFUCCI.
